# A Survey of the Use of Iterative Reconstruction Algorithms in Electron Microscopy

**DOI:** 10.1155/2017/6482567

**Published:** 2017-09-17

**Authors:** C. O. S. Sorzano, J. Vargas, J. Otón, J. M. de la Rosa-Trevín, J. L. Vilas, M. Kazemi, R. Melero, L. del Caño, J. Cuenca, P. Conesa, J. Gómez-Blanco, R. Marabini, J. M. Carazo

**Affiliations:** ^1^Centro Nacional de Biotecnología, CSIC, c/Darwin 3, Cantoblanco, 28049 Madrid, Spain; ^2^Universidad CEU San Pablo, Campus Urbanizacion Montepríncipe, Boadilla del Monte, 28668 Madrid, Spain; ^3^Universidad Autónoma de Madrid, Cantoblanco, 28049 Madrid, Spain

## Abstract

One of the key steps in Electron Microscopy is the tomographic reconstruction of a three-dimensional (3D) map of the specimen being studied from a set of two-dimensional (2D) projections acquired at the microscope. This tomographic reconstruction may be performed with different reconstruction algorithms that can be grouped into several large families: direct Fourier inversion methods, back-projection methods, Radon methods, or iterative algorithms. In this review, we focus on the latter family of algorithms, explaining the mathematical rationale behind the different algorithms in this family as they have been introduced in the field of Electron Microscopy. We cover their use in Single Particle Analysis (SPA) as well as in Electron Tomography (ET).

## 1. Introduction

Electron Microscopy has been established as one of the key players in Structural Biology with the goal of elucidating the three-dimensional structure of macromolecular complexes in order to better understand their function and molecular mechanisms [1–3]. One of the most important steps in the image processing pipeline is the 3D reconstruction of a map compatible with the projections acquired at the microscope [4]. In practice, projections of the macromolecule are contaminated by a huge amount of noise (typical Signal-to-Noise Ratios in the order of 0.01; i.e., there is 100 times more noise power than signal power, Hosseinizadeh et al. [5]), and the 3D reconstruction emerges, in a simplified way, as the 3D “average” of thousands of projections, each one looking at the molecule from a different point of view [4]. In the 3D space, the signal coming from the macromolecules is reinforced by the averaging process. However, random noise tends to be canceled by this averaging. Currently, the 3D reconstruction step is no longer seen as a limiting step (except for its execution time) in Single Particle Analysis due to the large number of particles involved in the reconstruction (between tens and hundreds of thousands), and direct Fourier inversion methods are currently the standard de facto [6–8]. These latter methods are especially well suited to handle a large number of projections thanks to their computational speed and their accuracy when the angular coverage of the set of projections fully fills the 3D Fourier space, which is currently normally the case in SPA (a word of caution should be expressed in those cases in which subsequent rounds of 3D classification significantly reduce the number of images per 3D class). However, in the past, there was an intense work of research in selecting the best reconstruction algorithm between four different families of reconstruction algorithms: (1) direct Fourier inversion [6–8], (2) back-projection algorithms [9–18], (3) iterative algorithms [19–43], and (4) Radon inversion methods [44–46].

The situation in Electron Tomography (ET) is, in general, specially involved, due to the combined effect of a smaller number of projection images, the existence of a missing wedge, and the fact that images are very large, posing an important challenge to the computational resources (especially in terms of memory; for a comparison between Single Particle Analysis and Electron Tomography, see Jonic et al. [47]). The research of 3D reconstruction algorithms trying to reduce reconstruction artifacts and the execution time has been rather intense, especially in the last decade [48–61]. Similarly, pure reconstruction algorithms in ET may be combined with a priori information incorporating the fact that the reconstruction is sparse in some domain or exploiting the discrete nature of the object being imaged [62–76].

In this review, we focus on the family of iterative reconstruction algorithms, also known as series expansion methods. The classical algorithms (ART, Block ART, and SIRT) have been followed by a number of more modern algorithms like Conjugate Gradient, Subgradient Descent, Projected Subgradient, Superiorization, ADMM, and so forth, allowing all kinds of regularizations with a special emphasis on sparsity promoting regularizers. We start by showing how the 3D reconstruction problem can be posed as a problem of solving a linear system of equations. Then, we put the more classical methods in a common algebraic framework. Finally, we introduce the rationale behind the more modern methods.

## 2. 3D Reconstruction as an Equation System Problem

Images collected by the electron microscope can be understood as the parallel projection of the Coulomb potential of the macromolecule being imaged. The relationship between the 3D model, $V(\tilde{r}):{\mathbb{R}}^{3}\times 1\to \mathbb{R}$, and a projection image, $I(\tilde{s}):{\mathbb{R}}^{2}\times 1\to \mathbb{R}$, is given by [77]

(1) $$\begin{array}{c} I_{\tilde{A}}\left(\;\tilde{s}\;\right)=\overset{\infty }{\underset{-\infty }{\int }}V\left(\;{\tilde{A}}^{-1}{\tilde{H}}^{T}\tilde{s}\;\right)dt, \end{array}$$

where $\tilde{r}=(x,y,z,1)$ and $\tilde{s}=(x,y,1)$ are the homogeneous coordinates of a 3D point and a 2D point, respectively; ${\tilde{H}}^{T}$ is

(2) $$\begin{array}{c} {\tilde{H}}^{T}=\left(\begin{array}{ccc} 1 & 0 & 0\\ 0 & 1 & 0\\ 0 & 0 & t\\ 0 & 0 & 1 \end{array}\right), \end{array}$$

with *t* being the integration variable, and

(3) $$\begin{array}{c} \tilde{A}=\left(\begin{array}{cccc} r_{11} & r_{12} & r_{13} & t_{x}\\ r_{21} & r_{22} & r_{23} & t_{y}\\ r_{31} & r_{32} & r_{33} & 0\\ 0 & 0 & 0 & 1 \end{array}\right)=\left(\begin{array}{cc} R & t\\ 0^{T} & 1 \end{array}\right) \end{array}$$

is the matrix that specifies the point of view from where the projection has been taken (*R* is a rotation matrix normally specified by 3 Euler angles and **t** is an in-plane displacement). Every projection has its own matrix $\tilde{A}$ reflecting the different projection directions and individual shifts of each image.

In practice, this ideal image is never observed but it is corrupted by random noise (whose nature is related to the structure of the ice surrounding the macromolecule, the random arrival of electrons, etc.) and the projected image is not known at any position $\tilde{s}$ but at a discrete set of positions (${\tilde{s}}_{i}$, normally the centers of the pixels of the acquired image). In this way, we may rewrite the above equation as

(4) $$\begin{array}{c} I_{\tilde{A},i}=I_{\tilde{A}}\left(\;{\tilde{s}}_{i}\;\right)=\overset{\infty }{\underset{-\infty }{\int }}V\left(\;{\tilde{A}}^{-1}{\tilde{H}}^{T}{\tilde{s}}_{i}\;\right)dt+n_{i}. \end{array}$$

Note that this image formation model uses additive noise, since the main source of noise is not the low number of electron counts at each pixel but the structure of the amorphous ice embedding the molecule. This noise has a Gaussian distribution as was shown in [78].

Let us assume now that we express the volume as a linear combination of functions, $b(\tilde{r})$, shifted to a set of known positions ${\tilde{r}}_{j}$ (e.g., usually these positions are regularly distributed on a grid [30]):

(5) $$\begin{array}{c} V\left(\;\tilde{r}\;\right)=\underset{j}{\sum }x_{j}b\left(\;\tilde{r}-{\tilde{r}}_{j}\;\right). \end{array}$$

The basis functions may be voxels or any other function with some interesting property from the tomographic point of view (for instance, Kaiser-Bessel modified functions, also known as blobs, [32, 36] are known to reduce the noise in the 3D reconstruction).

The goal of the tomographic problem is to determine the basis coefficients, *x*_*j*_, such that the experimental projection is actually the line integral of the volume *V*. Substituting (5) into (4) and disregarding the noise term (it will be recovered later), we have

(6) IA~,i=∫−∞∞VA~−1H~Ts~idt=∫−∞∞∑jxjbA~−1H~Ts~i−r~jdt=∑jxj∫−∞∞bA~−1H~Ts~i−r~jdt=∑jxjaA~,ij,

where $a_{\tilde{A},ij}$ is the line integral of the basis function located at ${\tilde{r}}_{j}$ at the location ${\tilde{s}}_{i}$ along the direction determined by the geometrical transformation $\tilde{A}$, that is, the contribution of the *j*-th basis function of the volume to the *i*-th pixel in the image. We may write the image pixels in a single vector using lexicographical order and we may rewrite the above equation in a matrix form:

(7) $$\begin{array}{c} I_{\tilde{A}}=A_{\tilde{A}}x. \end{array}$$

If we have images of size *P* × *P* pixels and assuming that the volume is reconstructed in a cube of size *B* × *B* × *B*, then $I_{\tilde{A}}\in {\mathbb{R}}^{P^{2}}$ is a vector in a space of dimension *P*^2^, **x** ∈ *ℝ*^*B*^3^^ is a vector in a space of dimension *B*^3^, and $A_{\tilde{A}}$ is a matrix of size *P*^2^ × *B*^3^. If we look again at the pixel level, we may write

(8) $$\begin{array}{c} I_{\tilde{A},i}=a_{\tilde{A},i}^{T}x=\langle \;a_{\tilde{A},i},x\;\rangle , \end{array}$$

where $a_{\tilde{A},i}^{T}$ is the *i*-th row of the matrix $A_{\tilde{A}}$. This is the equation of a hyperplane in the *ℝ*^*B*^3^^ space, meaning that every single pixel of the projection image provides a hyperplane constraint for locating the volume coefficients **x**. The true **x**, in the ideal noiseless case, must be at the intersection of all the hyperplanes defined by all pixels (the noise observed in the measurements simply randomly shifts each hyperplane along the direction perpendicular to the hyperplane normal). In this way, a single projection provides *P*^2^ equations, but we have *B*^3^ unknowns so that there is an infinite number of structures compatible with a single projection. In Electron Microscopy (EM), we collect thousands of projection images (let us say *M*) and we may stack all the measurements in a big vector. The corresponding equation system would then be

(9) $$\begin{array}{c} \left(\begin{array}{c} I_{{\tilde{A}}_{1}}\\ I_{{\tilde{A}}_{2}}\\ \vdots \\ I_{{\tilde{A}}_{M}} \end{array}\right)=\left(\begin{array}{c} A_{{\tilde{A}}_{1}}\\ A_{{\tilde{A}}_{2}}\\ \vdots \\ A_{{\tilde{A}}_{M}} \end{array}\right)x \end{array}$$

which in general can be written as the linear equation system

(10) $$\begin{array}{c} b=Ax, \end{array}$$

where **b** ∈ *ℝ*^*MP*^2^^ is a vector collecting all the experimental images and *A* is a matrix of size *MP*^2^ × *B*^3^ collecting all the projection matrices. Note that this matrix is very sparse since, for common basis functions like voxels, blobs, and so forth, just a few coefficients in the volume contribute to each pixel (only those coefficients along the integration line passing through that pixel). Just to give some typical numbers in EM, Let us say we have *M* = 50,000 images of size *P* × *P* = 256 × 256. We would have *MP*^2^ = 3.3 billion equations and *B*^3^ = *P*^3^ = 16.8 million unknowns (if voxels are used as basis function). The equation system is inconsistent because of the measurement noise and some kind of least squares solution must be sought [79]. We will do so in the next section.

We have presented the linear equation system only in connection to the data collection geometry. However, the Contrast Transfer Function (CTF) of the microscope (i.e., how the microscope blurs the ideal images) can easily be incorporated. The convolution can be represented by a matrix multiplication using a Toeplitz matrix, such that instead of the $A_{\tilde{A}}$ matrices that contain purely geometrical information we may use the matrices $CA_{\tilde{A}}$, where *C* is the CTF Toeplitz matrix (each experimental image may have its own CTF matrix).

Interestingly, posing the tomographic problem as a linear equation system can also be done in Fourier space. Thanks to the Central Slice Theorem, the relationship between the 3D Fourier transform of the macromolecule model and the 2D Fourier transform of the projection can be expressed as [77]

(11) $$\begin{array}{c} {\hat{I}}_{\tilde{A}}\left(\;\tilde{S}\;\right)=e^{-i\langle \tilde{S},H_{0}Rt\rangle }\hat{V}\left(\;{\tilde{A}}^{\prime T}{\tilde{H}}_{0}^{T}\tilde{S}\;\right), \end{array}$$

where ${\tilde{A}}^{\prime }=\left(\begin{array}{cc} R & 0\\ 0^{T} & 1 \end{array}\right)$, ${\tilde{H}}_{0}$ is the matrix defined at (2) with *t* = 0, and *H*_0_ is the same matrix except the last row (the one adding its homogeneous nature). $\tilde{S}$ is the 2D frequency coordinate and ${\hat{I}}_{\tilde{A}}$ and $\hat{V}$ are the 2D and 3D Fourier transforms of the projection image and the macromolecular model, respectively. Equation (11) means that, to evaluate the Fourier coefficient at the 2D frequency coordinate $\tilde{S}$, we need to evaluate the Fourier transform of the volume at the coordinate ${\tilde{A}}^{\prime T}{\tilde{H}}_{0}^{T}\tilde{S}$ and then multiply by the corresponding phase term, $e^{-i\langle \tilde{S},H_{0}Rt\rangle }$, to account for the shift in the images. In practice, we do not have the Fourier transform of the volume at any possible location. We will have to interpolate its value from the 3D Fourier coefficients in its surroundings. Let us concentrate at the frequency ${\tilde{S}}_{j}$ and let us call the corresponding 2D Fourier coefficient ${\hat{I}}_{j}$. Let us refer to the *j*-th 3D Fourier coefficient by ${\hat{V}}_{j}$. Then, we may interpolate the 2D Fourier coefficient as

(12) $$\begin{array}{c} {\hat{I}}_{i}=\underset{j}{\sum }{\hat{V}}_{j}{\hat{a}}_{\tilde{A},ij}, \end{array}$$

where the terms ${\hat{a}}_{\tilde{A},ij}$ comprise the phase shift as well as the interpolation weights. This equation is formally identical to (6), meaning that the 3D reconstruction problem in Fourier space can also be expressed as a linear system of equations, and we have to be careful to perform the complex interpolation. Actually, this is the position taken in Scheres [80] and Chen and Förster [59]. Including the CTF in this framework is even easier than in the real space case, since it suffices to multiply the ${\hat{a}}_{\tilde{A},ij}$ terms by the *c*_*i*_ CTF coefficients.

## 3. (Weighted) Least Squares Solution

Given the inconsistent equation system *A ***x** = **b**, we may try to look for the **x** that minimizes its distance to all the hyperplanes given by the experimentally measured pixel values. This is achieved by minimizing the norm of the residuals vectors *A ***x** − **b**. In tomographic terms, *A ***x** may be interpreted as the expected experimental projections given a macromolecular model **x**, while **b** are the experimentally measured projections. The idea is to find the model **x** such that its reprojections are as similar as possible to the experimentally acquired images. The residual of the comparison between the experimental images and the reprojections should be just the noise present in the experimental images:

(13) $$\begin{array}{c} x=\underset{x}{\arg\min}{\left\|\;Ax-b\;\right\|}^{2}, \end{array}$$

where the norm is calculated using the standard inner product:

(14) $$\begin{array}{c} {\left\|\;u\;\right\|}^{2}=\langle \;u,u\;\rangle =u^{T}u. \end{array}$$

This is a linear least squares problem. Note that the solution is not unique (any vector **x**_0_ in the kernel of the matrix *A* will yield the same error norm, because *A*(**x** + **x**_0_) = *A ***x** + 0 = *A ***x**). In practice, **x**_0_ refers to any structure whose Fourier coefficients are in areas not measured by the experimental images. Developing the norm in the optimization problem, we have

(15) x=arg⁡minx⁡Ax−bTAx−b=arg⁡minx⁡b2+xTATAx−2xTATb.

If we now differentiate and equate to 0, we have

(16) $$\begin{array}{c} \frac{\partial {\left\|Ax-b\right\|}^{2}}{\partial x}=2A^{T}Ax-2A^{T}b=0 \end{array}$$

or equivalently

(17) $$\begin{array}{c} A^{T}Ax=A^{T}b. \end{array}$$

This latter equation is known as the normal equation of the least squares problem. It can be shown that any solution of the normal equations is also a solution of the least squares problem, and, conversely, any solution of the least squares problem must also be a solution of the normal equations [81, Chapter 6]. Except for degenerate cases, *A*^*T*^*A* is a positive definite matrix, implying that the matrix is invertible, and consequently the normal equations have a unique solution. For complex numbers, the norm is defined as

(18) $$\begin{array}{c} {\left\|\;u\;\right\|}^{2}=u^{H}u \end{array}$$

with *H* being the conjugate transpose, so that the normal equations in Fourier space would be

(19) $$\begin{array}{c} A^{H}Ax=A^{H}b. \end{array}$$

We may now introduce weights in the minimization if there are some measurements that are more reliable than others (in real space, this is more difficult, but in Fourier space, low frequency components are overrepresented with respect to high frequency ones and they are consequently downweighted, Chen and Förster [59]). Given a diagonal, positive definite matrix *W* of size *MP*^2^ × *MP*^2^, we may define the *W* inner product as

(20) $$\begin{array}{c} {\langle \;u,v\;\rangle }_{W}=u^{H}Wv. \end{array}$$

The normal equations become in this case

(21) $$\begin{array}{c} A^{H}WAx=A^{H}Wb. \end{array}$$

The most straightforward approach to solving this equation system is the use of the Moore-Penrose pseudoinverse:

(22) $$\begin{array}{c} x={\left(\;A^{H}WA\;\right)}^{-1}A^{H}Wb=A^{†}b. \end{array}$$

The matrix *A*^*H*^*WA* is of size *B*^3^ × *B*^3^ and its direct inversion is normally out of the reach of any current computer. Numerical algorithms that avoid the need to invert this matrix are normally used and they are explained in the next section.

## 4. Iterative Solutions of a Linear Equation System

Note that all equation systems posed so far (see (6), (12), (17), (19), and (21)) are of the generic form:

(23) $$\begin{array}{c} Ax=b. \end{array}$$

In this section, *𝒜* and *b* are the generic system matrix and data terms, independently of their actual expressions in terms of the microscopic projection images, CTFs, Fourier or real space versions, and so forth, since these details have already been carefully presented in the sections above.

A very simple, but general, approach to produce an iterative algorithm solving the equation system in (23) is to decompose matrix *𝒜* as the difference of two other matrices, *ℳ* and *𝒩*, as in

(24) $$\begin{array}{c} A=M-N. \end{array}$$

Then,

(25) M−Nx=bMxk+1=Nxk+bxk+1=M−1Nxk+M−1b=xk+M−1b−Axk.

This iterative scheme has the interesting property that if the residuals are *b* − *𝒜 ***x**^(*k*)^ = 0 (i.e., we have successfully found a solution of the equation system), then there is no update of the current solution.

*(i) Jacobi*. *𝒜* is decomposed in its diagonal matrix *𝒟* and two lower *ℒ* and upper *𝒰* triangular matrices:

(26) A=D−L+U⟹xk+1=xk+D−1b−Axk.

*(ii) Gauss-Seidel*. *𝒜* is decomposed in a similar way to the Jacobi decomposition above, but the *ℒ* matrix is used differently:

(27) A=L+D−U⟹xk+1=xk+L+D−1b−Axk.

*(iii) Richardson*. *𝒜* is decomposed using the identity matrix as reference:

(28) A=I−I−A⟹xk+1=xk+b−Axk.

The reasons why these numerical schemes succeed in solving the equation system *𝒜 ***x** = *b* can be nicely illustrated in Richardson's scheme. We may rewrite the iterative algorithm as

(29) $$\begin{array}{c} x^{\left(k+1\right)}=b+\left(\;I-A\;\right)x^{\left(k\right)}. \end{array}$$

We start with **x**^(0)^ = 0. Then, the estimates of **x** would be

(30) $$\begin{array}{c} x^{\left(1\right)}=b\\ x^{\left(2\right)}=b+\left(\;I-A\;\right)b\\ x^{\left(3\right)}=b+\left(\;I-A\;\right)b+{\left(\;I-A\;\right)}^{2}b\\ \vdots \\ x^{\left(k\right)}=\left(\;\overset{k-1}{\underset{m=0}{\sum }}{\left(\;I-A\;\right)}^{m}\;\right)b. \end{array}$$

If *𝒜* is an invertible matrix, then ∑_*m*=0_^*∞*^(*I* − *𝒜*)^*m*^ converges to *𝒜*^−1^ as long as for all eigenvalues of the matrix *𝒜*, *λ*_*i*_, it is fulfilled that 0 < |1 − *λ*_*i*_| < 1, which for real eigenvalues translates into 0 < *λ*_*i*_ < 2 [82, Section 3.5.1]. Actually, this family of iterative algorithms is normally modified to introduce a relaxation factor *μ*_*k*_ (which may be different for each iteration):

(31) $$\begin{array}{c} x^{\left(k+1\right)}=x^{\left(k\right)}+\mu_{k}M^{-1}\left(\;b-Ax^{\left(k\right)}\;\right). \end{array}$$

This relaxation factor helps to increase the radius of convergence of the algorithm by modifying the eigenvalues of the matrix involved in the convergence analysis (we have seen the analysis for Richardson's iteration but different matrices are needed for the rest of the schemes). There are works analyzing the convergence of the reconstruction algorithm in terms of the properties of the sequence of numbers *μ*_*k*_ [83].

SIRT is one of the most popular reconstruction algorithms used in Electron Tomography. As we show below, SIRT is the result of the Jacobi algorithm applied to the normal equations with a particular weighting scheme. Let us consider the normal equations of the Weighted Least Squares problem in real space:

(32) $$\begin{array}{c} A^{T}WAx=A^{T}Wb. \end{array}$$

The Jacobi update with relaxation parameter is

(33) $$\begin{array}{c} x^{\left(k+1\right)}=x^{\left(k\right)}+\mu_{k}D^{-1}\left(\;b-Ax^{\left(k\right)}\;\right). \end{array}$$

Let us decompose matrix *A* in its different rows (remember that the *i*-th row indicates how each basis function in the volume contributes to the *i*-th pixel),

(34) $$\begin{array}{c} A=\left(\begin{array}{c} a_{1}^{T}\\ a_{2}^{T}\\ \vdots \\ a_{MP^{2}}^{T} \end{array}\right), \end{array}$$

and columns (the *j*-th column indicates how the *j*-th basis function affects all the pixels in the measurements),

(35) $$\begin{array}{c} A=\left(\begin{array}{cccc} \alpha_{1} & \alpha_{2} & \cdots & \alpha_{B^{3}} \end{array}\right). \end{array}$$

Then,

(36) A=ATWA=∑i=1MP2wiaiaiTb=ATWb=∑i=1MP2wibiaiAxk=∑i=1MP2wiaiaiTxk=∑i=1MP2wi∑m=1B3aimxmkai.

Let us now concentrate on a given basis function *j*:

(37) djj=∑i=1MP2wiaij2=αjW2bj=∑i=1MP2wibiaij=αj,bWAxkj=∑i=1MP2wi∑m=1B3aimxmkaij=αj,AxkWxjk+1=xjk+μkdjj−1bj−Axkj=xjk+μkαj,b−AxkWαjW2.

Interestingly, if the current image residual (**b** − *𝒜 ***x**^(*k*)^) is *W*-orthogonal to **α**_*j*_, that is, changing the coefficient of the *j*-th basis function does not affect the residual, then, as expected, the coefficient of the *j*-th basis function is not changed.

In EM, we are used to formulate SIRT as

(38) $$\begin{array}{c} x^{\left(k+1\right)}=x^{\left(k\right)}+\mu_{k}\overset{MP^{2}}{\underset{i=1}{\sum }}w_{i}\frac{b_{i}-\langle a_{i},x^{\left(k\right)}\rangle }{{\left\|a_{i}\right\|}^{2}}a_{i}. \end{array}$$

In the following, let us show that both formulations (see (37) and (38)) are not equivalent. Actually SIRT is not a single algorithm but a full family of reconstruction algorithms [84]; each one provides a different insight into the reconstruction process. If we look at a particular basis function *j* in (38), we have

(39) xjk+1=xjk+μk∑i=1MP2wibi−ai,xkai2aij=xjk+μk∑i=1MP2wiai2bi−ai,xkaij.

Let us now work on (37):

(40) xjk+1=xjk+μkdjj−1bj−Axkj=xjk+μk·1αjW2∑i=1MP2wibiaij−∑i=1MP2wi∑m=1B3aimxmkaij=xjk+μk1αjW2∑i=1MP2wibi−ai,xkaij.

The algorithms in (39) and (40) are clearly different and, however, both are called SIRT and both belong to the SIRT family of algorithms. Any algorithm of the form

(41) $$\begin{array}{c} x_{j}^{\left(k+1\right)}=x_{j}^{\left(k\right)}+\mu_{k}\frac{1}{\gamma_{j}}\overset{MP^{2}}{\underset{i=1}{\sum }}\frac{w_{i}}{\rho_{i}}\left(\;b_{i}-\langle \;a_{i},x^{\left(k\right)}\;\rangle \;\right)a_{ij} \end{array}$$

for suitable *γ*_*j*_ and *ρ*_*i*_ numbers is also considered a SIRT algorithm [84]. A particular class for which convergence has been proven [84] is for

(42) γj=∑i=1MP2wiaijαρi=∑j=1B3aij2−α0<α≤2,  0<μk<2.

The Jacobi iteration resulting in (40) corresponds to *α* = 2; the typical EM SIRT (see (39)) corresponds to *α* = 0 and is the one implemented in Xmipp [85, 86] and TomoJ [87]. However, ASTRA toolbox [88], also used in EM, has a SIRT algorithm with *α* = 1.

The case where *α* = 0 (see (38)) makes an interesting connection to the theory of feasibility problems [89–91]. Consider the hyperplane defined by all volumes compatible with the *i*-th experimental measurement:

(43) $$\begin{array}{c} H_{i}=\left\{\;x\in R^{B^{3}}\;|\;\langle \;a_{i},x\;\rangle =b_{i}\;\right\}. \end{array}$$

Solving the tomographic problem amounts to finding a volume **x** such that it is compatible with all measurements (note that, in this formulation, we are disregarding the effect of noise which makes such an intersection be empty):

(44) $$\begin{array}{c} x\in H_{1}\cap H_{2}\cap \cdots \cap H_{MP^{2}}. \end{array}$$

Given a volume **x**^(*k*)^, we may orthogonally project it onto the hyperplane given by the *i*-th measurement by

(45) $$\begin{array}{c} Proj_{H_{i}}\left\{\;x^{\left(k\right)}\;\right\}=x^{\left(k\right)}+\frac{b_{i}-\langle a_{i},x^{\left(k\right)}\rangle }{{\left\|a_{i}\right\|}^{2}}a_{i}. \end{array}$$

In this way, we may rewrite the SIRT iteration with *α* = 0 as

(46) $$\begin{array}{c} x^{\left(k+1\right)}=x^{\left(k\right)}+\mu_{k}\overset{MP^{2}}{\underset{i=1}{\sum }}w_{i}\left(\;Proj_{H_{i}}\left\{\;x^{\left(k\right)}\;\right\}-x^{\left(k\right)}\;\right). \end{array}$$

That is, at every iteration, we update the volume with a weighted sum of the orthogonal projections of the current solution onto the set of hyperplanes defined by the experimental measurements. The relaxation factor *μ*_*k*_ (normally chosen between 0 and 1, although there are convergence theorems for values between 0 and 2) could be understood as how much we go from our current position **x**^(*k*)^ to the desired position Proj_*H*_*i*__{**x**^(*k*)^}. In a noiseless case, we are rather certain about the update and may set *μ*_*k*_ = 1. In a noisy environment, we may be more conservative and use a smaller relaxation factor reflecting our distrust in the experimental measurements.

Actually, we may update our estimate of the current solution after a single hyperplane projection; that is, we do not have to wait to “see” all measurements at the same time but we may update the volume just after seeing each pixel value:

(47) $$\begin{array}{c} x^{\left(k+1\right)}=x^{\left(k\right)}+\mu_{k}w_{i\left(k\right)}\left(\;Proj_{H_{i\left(k\right)}}\left\{\;x^{\left(k\right)}\;\right\}-x^{\left(k\right)}\;\right). \end{array}$$

The index *i*(*k*) will go over all the experimental measurements. This scheme is known as ART (algebraic reconstruction technique) and the difference between SIRT and ART is the same as the difference between a Jacobi update (SIRT) in the solution of a linear equation system and a Gauss-Seidel update (ART). In the ART case, we update the volume as soon as we have new information available, while in the SIRT case, we update the volume with a consensus of all the information available. This property of ART can be exploited to use ART in a streaming mode as data is acquired and stop data acquisition as soon as the reconstruction achieves a specific criterion (this could minimize beam damage, for instance) [92]. ART converges much faster than SIRT, although it tends to produce little bit noisier reconstructions. This problem can be alleviated through the choice of a relaxation factor that decreases over time. A trade-off between updating after seeing every pixel (ART) and updating after seeing all pixels (SIRT) is given by Block-ART or Simultaneous ART (SART). The volume is updated after seeing a small set of pixels *S*_*k*_ (normally all those in the same experimental image):

(48) $$\begin{array}{c} x^{\left(k+1\right)}=x^{\left(k\right)}+\mu_{k}\underset{i\in S_{k}}{\sum }w_{i}\left(\;Proj_{H_{i}}\left\{\;x^{\left(k\right)}\;\right\}-x^{\left(k\right)}\;\right). \end{array}$$

Kunz and Frangakis [73] studied how the order in which these blocks were chosen affected the reconstruction's quality.

Additionally, we do not need to be restricted to orthogonal projections, and oblique projections can be undertaken. Given a symmetric, positive definite matrix *G*, the oblique projection onto the hyperplane *H*_*i*_ is defined as [93]

(49) $$\begin{array}{c} Proj_{H_{i}}^{G}\left\{\;x^{\left(k\right)}\;\right\}=x^{\left(k\right)}+\frac{b_{i}-\langle a_{i},x^{\left(k\right)}\rangle }{{\left\|a_{i}\right\|}_{G^{-1}}^{2}}G^{-1}a_{i}. \end{array}$$

The study of these alternatives has given rise to a whole family of iterative algorithms (Cimmino, Component Averaging (CAV), Block-iterative CAV (BiCAV), Block-Simplified SART, iterative algorithms with Bregman projections, Block-iterative Underrelaxed Entropy Projections, Averaging Strings, etc.) [93–98]. A different variant of ART is a multiplicative ART (MART), where the iterative step is multiplicative instead of additive. Although some of these algorithms have been tested in Electron Microscopy [40, 99], none of these more advanced variants have made their way into a massive adoption. For extensive reviews of the algebraic reconstruction techniques, the reader is referred to Gordon and Herman [100], Gordon [101], and Herman [102].

All SIRT algorithms can be written in a compact matrix form:

(50) $$\begin{array}{c} x^{\left(k+1\right)}=x^{\left(k\right)}+\mu_{k}CA^{T}R\left(\;b-Ax^{\left(k\right)}\;\right), \end{array}$$

with *A* being the system matrix (*A* projects the current estimate of the solution onto the image projection space, while *A*^*T*^ back-projects the residual into the volume space) and *R* and *C* being suitable diagonal matrices (*C* acting on the basis function space *c*_*jj*_ = 1/*γ*_*j*_; and *R* acting on the experimental measurements space *r*_*ii*_ = *w*_*i*_/*ρ*_*i*_).

Stated in this matrix form, we may easily find another SIRT algorithm that is very popular in image processing: the Landweber iteration. A possible solution to the Weighted Least Squares problem,

(51) x=arg⁡minx⁡Ex=12Ax−bW2=arg⁡minx⁡12Ax−bTWAx−b=arg⁡minx⁡12bW2+12xTATWAx−xTATWb,

is to use gradient descent iterations:

(52) xk+1=xk−μk∇Exk=xk−μkATWAx−ATWb=xk+μkATWb−Ax.

This latter iteration is called Landweber iteration and it can be easily recognized that it fits into the generic SIRT form of (50).

Another interesting variant of this family of traditional algorithms is the possibility to use unmatched projectors, for example, a relatively complicated forward operator, *A*, including the projection geometry and multiple defoci effects and a simple backward operator, *B*, considering only the projection geometry [103]:

(53) $$\begin{array}{c} x^{\left(k+1\right)}=x^{\left(k\right)}+\mu_{k}CB^{T}R\left(\;b-Ax\;\right). \end{array}$$

The convergence of this kind of algorithms depends on the eigenvalues of the matrix *I* − *CB*^*T*^*RA* and the specific details can be found in [103]. In EM, this strategy has been implemented in Xmipp (the forward projection considers geometry and CTF, but the backward projection only considers geometry). Although it was shown to successfully recover the underlying 3D structure, this method has not been further pursued because of the general problem of most iterative algorithms: their computational speed compared to direct Fourier inversion algorithms.

In recent years, one of the most popular algorithms for solving a linear equation system is Conjugate Gradient [59, 104–106]. The success of this algorithm is demonstrated in its ability to converge to the local solution in *B*^3^ steps (as many steps as the dimension of the solution being sought). Note that this number is finite and much smaller than the number of steps of ART or SIRT, whose convergence theorems are on the limit as the number of iterations goes to infinity. Given the equation system

(54) $$\begin{array}{c} Ax=b, \end{array}$$

the trick is to use a set of directions {**p**_*k*_} which is orthogonal with respect to the inner product induced by *𝒜* (*k* ≠ *k*′⇒〈**p**_*k*_, **p**_*k*′_〉_*𝒜*_ = 0; these directions are said to be conjugate with respect to *𝒜*). Then the solution sought can be written in the form

(55) $$\begin{array}{c} x^{\left(k\right)}=\overset{k}{\underset{k^{\prime }=0}{\sum }}\frac{\langle p_{k^{\prime }},b\rangle }{{\left\|p_{k^{\prime }}\right\|}_{A}^{2}}p_{k^{\prime }}. \end{array}$$

The set of conjugate directions is also iteratively constructed as

(56) $$\begin{array}{c} p_{k+1}=\left(\;b-Ax^{\left(k\right)}\;\right)-\overset{k}{\underset{k^{\prime }=0}{\sum }}\frac{\langle p_{k^{\prime }},b-Ax^{\left(k\right)}\rangle }{{\left\|p_{k^{\prime }}\right\|}_{A}^{2}}p_{k^{\prime }}. \end{array}$$

Actually, these summations can be nicely reorganized so that in real implementation we only need to keep a vector for the current solution, a vector with the residuals, and a vector with the current conjugate direction [104].

In the context of EM, the Conjugate Gradient was used by Chen and Förster [59] in Fourier space. However, there are a number of variants to the basic Conjugate Gradient algorithm (Conjugate Residuals, Biconjugate Gradient, Stabilized Biconjugate Gradient, Lanczos method, Generalized Minimal Residuals (GMINRES), Bi-Lanczos, Conjugate Gradient Squared, Quasi Minimal Residuals, etc.; most of them belong to a family of algorithms called Krylov subspace algorithms [107, 108]), none of which have been tested in EM.

In practice, none of these iterative algorithms are run to convergence. Instead, the algorithms are typically run for a fixed number of iterations (typically *M* or 2*M* in the case of Block ART, 20 for CG, and 100–150 in the case of SIRT). How deep these algorithms have gone in the objective function landscape depends on the conditioning number of the equation system:

(57) $$\begin{array}{c} \kappa \left(\;A\;\right)=\frac{\sigma_{max}}{\sigma_{min}}, \end{array}$$

where *σ*_max_ and *σ*_min_ are the maximum and minimum singular values of matrix *𝒜*. If this ratio is close to 1, iterative algorithms will quickly converge to the minimum of the error function. If the ratio is much larger than 1, then the problem is said to be ill-posed (small perturbations in the *b* vector may translate into large variations in the solution vector **x**) and the convergence speed of iterative algorithms is slow. In case of solving normal equations, we have

(58) $$\begin{array}{c} \kappa \left(\;A\;\right)=\kappa \left(\;A^{T}A\;\right)=\kappa^{2}\left(\;A\;\right). \end{array}$$

That is, the ill-posed character of matrix *A* is worsened. For this reason, in the theory of linear equation solving, it is customary to use a preconditioning matrix *P* so that we do not solve the problem

(59) $$\begin{array}{c} Ax=b \end{array}$$

but

(60) $$\begin{array}{c} P^{-1}Ax=P^{-1}b. \end{array}$$

The preconditioning *P* matrix is chosen in such a way that

(61) $$\begin{array}{c} \kappa \left(\;P^{-1}A\;\right)<\kappa \left(\;A\;\right). \end{array}$$

Although several preconditioners have been tested in different kinds of tomographic setups (computerized tomography [109], optical diffusion tomography [110], positron emission tomography [111], electrical capacitance tomography [112], acoustic waveform tomography [113], etc.), in EM, there has not been any attempt to use any preconditioning, although this is an issue the community is aware of [114].

## 5. Constrained 3D Reconstruction

The feasibility problem of (44) can be complemented with some additional constraints representing a priori knowledge about the reconstruction. We may do so by imposing the fact that the volume also belongs to some convex set (a set is convex if, for any two volumes in this set, **x**_1_ and **x**_2_, the linear combination (1 − *λ*)**x**_1_ + *λ ***x**_2_, with 0 ≤ *λ* ≤ 1, also belongs to that set; the set of all nonnegative volumes is convex as well as the set of all volumes defined within a mask, the set of all volumes with a given symmetry, the set of all volumes bandlimited to a given frequency, etc. [64]). Given a collection of convex sets representing our a priori knowledge about the reconstructed particle, *C*_1_, *C*_2_,…, *C*_*K*_, we may try to find a feasible volume at the intersection

(62) $$\begin{array}{c} x\in H_{1}\cap H_{2}\cap \cdots \cap H_{MP^{2}}\cap C_{1}\cap C_{2}\cap \cdots \cap C_{K}. \end{array}$$

This problem has been extensively explored in the EM community under the name Projection Onto Convex Sets (POCS) and the interested reader is referred to Carazo and Carrascosa [115, 116], Carazo [117], García et al. [31], Sorzano et al. [64], and Deng et al. [118]. The idea is to alternate between projections onto the subspaces defined by the experimental measurements (*H*_*i*_) and projections onto the convex sets (the projector onto the set of nonnegative volumes simply sets all negative values to 0; the projector onto the set of volumes defined within a mask applies that mask to the current solution; the projector onto the set of symmetric volumes symmetrizes the current solution; the projector onto the set of bandlimited volumes applies a low-pass filter to the current solution; etc.). In the signal processing community, POCS algorithms have been generalized by algorithms using the so-called Proximity operators. Despite being out of the scope of this review, because these algorithms have not been introduced in EM, the interested reader may follow the review of Combettes and Pesquet [119] and the references therein.

Landweber iterations can also be set in a constrained setup. Let us assume that we have the a priori knowledge that **x** is in a convex set *C*. Then, the solution of the constrained problem

(63) $$\begin{array}{c} x=\underset{x\in C}{\arg\min}\frac{1}{2}{\left\|\;Ax-b\;\right\|}_{W}^{2} \end{array}$$

can be found with the iterative algorithm [119]:

(64) $$\begin{array}{c} x^{\left(k+1\right)}=Proj_{C}\left\{\;x^{\left(k\right)}+\mu_{k}A^{T}W\left(\;b-Ax\;\right)\;\right\}. \end{array}$$

As seen in the two examples above, one of the most interesting ideas of this constrained optimization is the possibility to alternate between the standard tomographic update (**x**^(*k*)^ + *μ*_*k*_*A*^*T*^*W*(**b** − *A ***x**)) and the projection onto convex sets (POCS, Proj_*C*_).

These ideas of constrained optimization can be further extended to nondifferentiable, convex functions (the *l*_1_ norm of the residual is a function of this kind). Let us assume that we are minimizing a nondifferentiable, convex function *ϕ*(**x**):

(65) $$\begin{array}{c} x=\arg\min\phi \left(\;x\;\right). \end{array}$$

At a differentiable point of *ϕ*, a gradient descent iteration is perfectly suitable to find the minimum of the objective function:

(66) $$\begin{array}{c} x^{\left(k+1\right)}=x^{\left(k\right)}-\mu_{k}\nabla \phi \left(\;x^{\left(k\right)}\;\right). \end{array}$$

The problem at a nondifferentiable point (which occurs normally at the frontiers of the intersection of convex sets) is that the gradient is not well defined. We may define instead the subgradient. A vector **g**_*k*_ ∈ *ℝ*^*B*^3^^ is a subgradient of *ϕ* at **x**^(*k*)^ if for any **x** we have

(67) $$\begin{array}{c} \phi \left(\;x\;\right)-\phi \left(\;x^{\left(k\right)}\;\right)\geq \langle \;g_{k},x-x^{\left(k\right)}\;\rangle . \end{array}$$

Intuitively, the gradient defines at differentiable points a unique hyperplane that is tangent to *ϕ* and, since *ϕ* is convex, this tangent plane is always below *ϕ*. At nondifferentiable points, there are an infinite number of hyperplanes touching at (**x**^(*k*)^, *ϕ*(**x**^(*k*)^)) and below *ϕ*. The normal vectors to these hyperplanes constitute the set of subgradients. The subgradient method iteration is then, at these nondifferentiable points [120, 121],

(68) $$\begin{array}{c} x^{\left(k+1\right)}=x^{\left(k\right)}-\mu_{k}g_{k}. \end{array}$$

We may easily add convex constraints to this method obtaining constrained subgradient minimization (known as Projected Subgradient minimization) [122, 123]:

(69) $$\begin{array}{c} x^{\left(k+1\right)}=Proj_{C}\left\{\;x^{\left(k\right)}-\mu_{k}g_{k}\;\right\}. \end{array}$$

Superiorization has been proposed as an alternative to Projected Subgradients [43, 124–126]. The idea of Superiorization is to steer the reconstruction iterations towards the constraints. One of the main differences between Projected Subgradient and Superiorization is that if there are several convex constraints, Projected Subgradient requires the projection onto the intersection of all of them, the feasible set, while Superiorization requires the sequential projection onto each of the constraints. Note that the intersection may be empty. For instance, the set of all volumes defined within a finitely supported mask and bandlimited is empty. Also, the projector onto the intersection set may be more complicated than the projector onto each one of the individual constraints. This limits the applicability of Projected Subgradient, while this is not a problem for Superiorization. Although these algorithms are available in the tomography community and are well characterized in other domains, none of them have made their way into EM.

A different approach to constrained optimization which has been actively explored in EM is by defining new equations that must be simultaneously solved along with the equation system coming from the measurements [64]. For instance, a mask can be easily expressed in terms of basis functions. Let us assume that there is no density coming from the molecule at a given location **r**_0_. Then, we may particularize the series expansion at **r**_0_ (see (5)) and add the linear equation

(70) $$\begin{array}{c} V\left(\;{\tilde{r}}_{0}\;\right)=\underset{j}{\sum }x_{j}b\left(\;{\tilde{r}}_{0}-{\tilde{r}}_{j}\;\right)=0. \end{array}$$

In this way, we handled a priori information coming from masks, symmetry, total molecular mass, and nonnegativity. Adding this a priori information was certainly relevant when the number of projections was small. However, as the number of projections increased, the improvement by the a priori information was less noticeable, showing that most of the information was already present in the experimental dataset. Now, with increasing pursuit of high-resolution results, there is again room for exploring these extra sources of information available, although for the moment this line of research has not been resumed.

Iteratively steering algorithms tend to promote reconstructions with certain characteristics. For instance, [65, 71, 72, 127, 128] focused on the problem of reconstructing discrete valued objects (the reconstructed volume could only have a few, normally two (background and foreground), values) or objects with very few active voxels. They use projectors similar to those used in convex sets; for instance, bgART [71] defined

(71) $$\begin{array}{c} Proj\left\{\;x_{j}^{\left(k\right)}\;\right\}=\left\{\begin{array}{cc} m_{k} & x_{j}^{\left(k\right)}<m_{k}+Ks_{k}\\ x_{j}^{\left(k\right)} & x_{j}^{\left(k\right)}\geq m_{k}+Ks_{k}, \end{array}\right. \end{array}$$

where *m*_*k*_ is the estimated mean of the background gray values at iteration *k*, *s*_*k*_ is their estimated standard deviation, and *K* is a user-selected multiple (normally a number between 3 and 6). The main difference between these iterative steering algorithms and those projecting onto convex sets is that the former project onto a nonconvex set; additionally, the set onto which the reconstruction is projected changes from iteration to iteration. The convergence of this kind of algorithms was studied in [129]. Reference [75] presented an algorithm for Electron Tomography in which the steering is driven by a nonlinear diffusion denoising algorithm. After each reconstruction step, the reconstructed volume is denoised by applying a step of a nonlinear diffusion algorithm (the projector). For these steering algorithms to converge, the reconstruction algorithm must be “perturbation resilient” [124] and the perturbation must be “small enough” as not to destroy the work of the reconstruction algorithm.

## 6. Sparse 3D Reconstructions

Sparse representations has been one of the most active research fields in the area of image and signal processing in the last 10–15 years [130–132]. The idea is that natural images and objects have a representation in some appropriate space in which very few nonnull coefficients are needed. This space may be fixed (e.g., wavelet transform or discrete cosine transform and DCT) or computed ad hoc for a particular problem (dictionary based algorithms). Knowing that our object has a sparse representation helps the algorithm to concentrate the energy in a few coefficients, preventing the energy dispersion normally caused by noise. In this problem setup, vector norms different from the Euclidean are normally employed. In general, the *l*_*p*_ norm is used:

(72) $$\begin{array}{c} {\left\|\;x\;\right\|}_{p}={\left(\;\sum_{j}{\left|\;x_{j}\;\right|}^{p}\;\right)}^{1/p}. \end{array}$$

Euclidean norm is obtained for *p* = 2, Manhattan norm is obtained for *p* = 1, and for *p* = 0 the norm of a vector is simply the count of the number of nonzero coefficients in the vector (technically, *l*_0_ is not a norm because it does not fulfill the condition ‖*λ ***x**‖_0_ = |*λ*|‖**x**‖_0_). The goal of sparsity is to find a representation of **x** such that

(73) x=arg min x0s.t. Dx=x,

where *D* is the dictionary of elements available to represent **x** (the wavelet, DCT, or ad hoc dictionary) and *x* is the representation of **x** in that dictionary. The aim is to have as few coefficients different from 0 as possible.

As stated above, sparse representations are mostly interesting in *l*_0_-norms. However, having this norm in the objective function requires combinatorial optimization techniques, known to be NP-hard in computational complexity. Interestingly, *l*_*p*_ norms with 0 < *p* < 2 tend to promote sparse representations (*x* has relatively few nonzero coefficients) and efficient algorithms have been developed in the recent years for these minimizations.

The first class of algorithms we will review involve an *l*_0_-norm and are those solving any of these two related problems:

(74) x=arg⁡min x0s.t. b−ADx22≤ϵ

(75) or  x=arg⁡min b−ADx22s.t. x0≤N.

Note that our “tomographic dictionary” (*𝒜D* = *A*^*T*^*AD*) includes the standard dictionary representation, *D*, as well as information about the projection structure of the tomographic setup *𝒜* = *A*^*T*^*A*. In the following, let us refer to this “tomographic dictionary” by *𝒟* and to its *j*-th column by *d*_*j*_ (in the dictionary jargon, this would be the *j*-th atom and we will assume that there are *J* ≪ *B*^3^ atoms in the dictionary). Many different algorithms exist to solve this problem [130]; none of them have been tested in EM. Among them, Matching Pursuit [133] and Orthogonal Matching Pursuit [134] are two of the most popular algorithms (actually the latter was tested in a tomography setting by researchers working on EM, although it was not applied to EM data [135]). For its simplicity, let us show the iterations in Matching Pursuit to illustrate the flavor of these algorithms and how they differ from the iterative algorithms presented so far.

Let *x*^(*k*)^ represent our current reconstruction. In the first iteration, it will be *x*^(0)^ = 0. We now choose the atom that is maximally aligned with the residual of our equation system, **r**^(*k*)^ (note that **r**^(0)^ = *b*), calculate its coefficient, and update the residual. This process is iterated until the desired number of atoms is reached. The following algorithm is executed from *k* = 0 to *k* = *N* − 1.

*(i) Step 1*. Seek the atom maximally aligned with this residual

(76) $$\begin{array}{c} j=\underset{j^{\prime }}{\arg\max}\left|\;\langle \;r^{\left(k\right)},d_{j^{\prime }}\;\rangle \;\right|. \end{array}$$

*(ii) Step 2*. Update the current solution with the projection of the residual onto this atom

(77) $$\begin{array}{c} x_{j^{\prime }}^{\left(k+1\right)}=\left\{\begin{array}{cc} x_{j^{\prime }}^{\left(k\right)} & j^{\prime }\neq j\\ \frac{\langle r^{\left(k\right)},d_{j}\rangle }{{\left\|d_{j}\right\|}^{2}} & j^{\prime }=j. \end{array}\right. \end{array}$$

*(iii) Step 3*. Update the residual

(78) $$\begin{array}{c} r^{\left(k+1\right)}=r^{\left(k\right)}-x_{j}^{\left(k+1\right)}d_{j}. \end{array}$$

This algorithm is greedy and finds a suboptimal solution of the reconstruction problem (see (75)) [133]. However, it is very easy to implement. A significant improvement is provided by Orthogonal Matching Pursuit in which Step 2 is modified to update all coefficients (not just one) by orthogonally projecting *b* onto the subspace spanned by all the atoms employed so far.

The second class of algorithms substitute the *l*_0_-norm by an *l*_1_-norm:

(79) x=arg⁡min x1s.t. b−ADx22≤ϵ

(80) or  x=arg⁡min b−ADx22s.t. x1≤δ.

This allows more efficient optimization algorithms to be employed. Classical algorithms are Least Absolute Shrinkage and Selection Operator (Lasso also known as Basis Pursuit), Iterative Reweighted Least Squares (IRLS), Iterative Shrinkage algorithms, Least Angle Regression (LARS), and any of their variants [130]. For their simplicity, Iterative Shrinkage algorithms have found their way into Electron Microscopy [136, 137] (for a theoretical background, you may also see [138, 139]). However, the EM versions are aimed at solving a regularized problem,

(81) $$\begin{array}{c} x=\arg\min{\left\|\;b-ADx\;\right\|}_{2}^{2}+\lambda {\left\|\;x\;\right\|}_{1}, \end{array}$$

rather than the constrained problems above. In their most simplified formulation, these algorithms alternate between a standard reconstruction update (let us, for instance, take a SIRT step) and a soft-thresholding step in some suitable, sparse space (e.g., the wavelet space). The rationale is relatively simple and resembles the line of thought of the iterative steering algorithms. After applying a standard reconstruction step, the solution is steered towards a sparse solution by setting to 0 all small coefficients in some space known to promote sparsity (as the wavelet space). A basic iterative step would be

(82) xk+1/2=xk+μkCATRb−Axkxk+1=∑j=1JSλxk+1/2,djdj,

where *S*_*λ*_(*x*) is the soft-thresholding function:

(83) $$\begin{array}{c} S_{\lambda }\left(\;x\;\right)=\left\{\begin{array}{cc} x+\frac{\lambda }{2} & x<-\frac{\lambda }{2}\\ 0 & \left|x\right|\leq \frac{\lambda }{2}\\ x-\frac{\lambda }{2} & x>\frac{\lambda }{2}. \end{array}\right. \end{array}$$

In the above iteration, we have assumed an orthonormal dictionary or transformation, but equivalent formulas can be found for nonorthonormal dictionaries.

The use of Lagrangian augmented objective functions of the form

(84) $$\begin{array}{c} x=\underset{x}{\arg\min}\;F\left(\;b-Ax\;\right)+\lambda G\left(\;x\;\right) \end{array}$$

is a widespread technique in data analysis. The first term, *F*, is called the data fidelity term, while the second term, *G*, is a regularizer. Many different schemes respond to this structure. For instance, a typical regularized Weighted Least Squares problem would be given by Tikhonov quadratic regularization (also known as ridge regression) involving some “preprocessing” matrix *L*,

(85) $$\begin{array}{c} x=\underset{x}{\arg\min}{\left\|\;b-Ax\;\right\|}_{W}^{2}+\lambda {\left\|\;Lx\;\right\|}_{2}^{2}, \end{array}$$

whose normal equations would be

(86) $$\begin{array}{c} \left(\;A^{T}WA+\lambda L^{T}L\;\right)x=A^{T}Wb. \end{array}$$

Assuming Gaussian errors for the measurements and a Gaussian prior for the volume coefficients, we would have *L* = Σ_**x**_^−1/2^.

(87) $$\begin{array}{c} \left(\;A^{T}WA+\lambda \Sigma_{x}^{-1}\;\right)x=A^{T}Wb. \end{array}$$

If we think that **x** and **b** are in Fourier space, these would be the normal equations associated with the same Bayesian problem that Relion is solving [7]. *W* contains the CTF information, *A* the data collection geometry (including the probability of each projection having a given projection direction and shift), and Σ_**x**_ our prior about the energy of the coefficients in Fourier space. A difference between Relion and these normal equations is that Relion reestimates the prior after each iteration.

We might have gone one step further to the generalized Tikhonov regularization,

(88) $$\begin{array}{c} x=\underset{x}{\arg\min}{\left\|\;b-Ax\;\right\|}_{W}^{2}+\lambda {\left\|\;L\left(\;x-x_{0}\;\right)\;\right\|}_{Q}^{2}, \end{array}$$

whose normal equations are

(89) $$\begin{array}{c} \left(\;A^{T}WA+\lambda L^{T}QL\;\right)x=A^{T}Wb+QLx_{0}. \end{array}$$

If we assume independence between the preprocessed coefficients, most maximum a posteriori (MAP) algorithms could be written using

(90) $$\begin{array}{c} G\left(\;Lx\;\right)=\underset{j}{\sum }\phi \left(\;{\left(\;Lx\;\right)}_{j}\;\right), \end{array}$$

where *ϕ*(*x*) is the a priori, negative log likelihood of observing a value of *x* (*ϕ*(*x*) = −log⁡*p*_**x**_(*x*)). Assume that a Gaussian distribution of the **x** coefficients results in *L* = Σ_**x**_^−1/2^ and *ϕ*(*x*) = *x*^2^ + *C* (*C* is a constant that does not affect the MAP optimization). Assume that a centered Laplace distribution of standard deviation *σ* ($p_{x}\left(x\right)=\left(1/\sqrt{2}\sigma \right)e^{-2|x|/\sqrt{2}\sigma }$) results in $L=\left(\sqrt{2}/\sigma \right)I$ and *ϕ*(*x*) = |*x*| + *C* with *G* being an *l*_1_-norm (instead of an *l*_2_-norm as in the case of the Gaussian prior). In an EM setup, Moriya et al. [74] assumed a Median Root Prior which favors locally monotonic reconstructions.

If we take *L* to be a volume derivative operator and take *G* to be the *l*_1_-norm of this derivative (that is, a Laplace prior on the derivative coefficients),

(91) $$\begin{array}{c} G\left(\;x\;\right)={\left\|\;Lx\;\right\|}_{1}, \end{array}$$

then we have a total variation regularization (also named as TVL1). The problem with the *l*_1_-norm is that it cannot be differentiated and sometimes it is substituted by

(92) Gx=∫R3∂Vr∂x2+∂Vr∂y2+∂Vr∂z2dr,

where *V*(**r**) is the volume reconstructed using the **x** coefficients (see (5)). This was explored for EM by Zhu et al. [62], Aganj et al. [63], Li et al. [66], Goris et al. [57], and Zhuge et al. [76].

The approach of Albarqouni et al. [140] also falls into this regularization category. The function *G* is, in their case, a function borrowed from robust statistics, the Huber function:

(93) $$\begin{array}{c} \phi_{\tau }\left(\;x\;\right)=\left\{\begin{array}{cc} 0.5x^{2} & \left|x\right|\leq \tau \\ \tau \left|x\right|-0.5\tau^{2} & \left|x\right|>\tau . \end{array}\right. \end{array}$$

This function is half-way between a quadratic and a linear penalization (*τ* controls the switch between these two behaviors). For low values of the derivatives, the function behaves as a quadratic term and for high values it behaves as a linear term. The idea is not to let large derivatives dominate the optimization process.

We can see that the above regularized problems include either some a priori knowledge on the volume (**x**) or a property of that volume (*L ***x**, normally its derivative). However, we could include both through a new algorithm (alternating-direction method of multipliers, ADMM) recently introduced for EM [141]:

(94) x=arg⁡min b−Ax22+λ1G1x+λ2G2us.t. u=Lx.

The actual problem being solved is an augmented Lagrangian:

(95) x=arg⁡minx,u,α⁡ Lμx,u,α=b−Ax22+λ1G1x+λ2G2u+αTLx−u+μ2Lx−u2,

where **α** is the set of Lagrangian multipliers. The ADMM proceeds iteratively as

(96) xk+1=arg⁡minx⁡ Lμx,uk,αkuk+1=arg⁡minu⁡ Lμxk+1,u,αkαk+1=αk+μLxk+1−uk+1.

Related to these sparse reconstruction problems is the one of compressed sensing. The idea is to perform a 2D or 3D reconstruction problem starting, not from a full image, but from a “few” incoherent points from the projection images [142–144]. The trick is that the a priori knowledge that the solution is sparse allows reconstructing the full image or volume from fewer points than Nyquist theorem requires (there is a lower limit on the number of measurements required). This theory has not been explored in biological EM applications, but it has been studied in material science EM [145–150], especially when Scanning TEM (STEM) is used [151]. For biological samples, Energy Filtered TEM (EFTEM) is a clear candidate to benefit from a compressed sensing acquisition [149].

## 7. Conclusions

The field of iterative reconstruction algorithms has been very much studied, particularly in its application to Electron Microscopy data, as we have shown in this review. The 3D reconstruction problem is no longer seen as a bottleneck in Single Particle Analysis (this is a technique in which many single particles, assumed to come from an homogeneous population but at different angular orientations, are combined into a single 3D map; Jonic et al. [47]), and the few attempts to complement the data with a priori knowledge are not in widespread use, probably due to their higher computational cost. However, as we approach resolutions of 2-3 Å, it may be worthy to retake this line of research as a way to increase the reconstruction resolution. In Electron Tomography, the situation is different due to the reconstruction artifacts induced by the maximum tilt angle limitation, the presence of gold beads, and the low number of projections. Additionally, the particular data collection geometry (normally, single tilt axis) favors the adoption of a pure 2D reconstruction approach that reduces the reconstruction problem in one dimension, implying a great reduction in the computational cost. In this field, iterative algorithms capable of incorporating a priori information are still a very active field of research. Traditional iterative algorithms as ART or SIRT still dominate the “market.” However, very powerful reconstruction algorithms with more modern approaches incorporating convex, nonconvex, and sparsity constraints are continuously appearing and most likely, in the near future, one of these algorithms will eventually become the standard de facto.
